# Deep Learning Model for Predicting Neurodevelopmental Outcome in Very Preterm Infants Using Cerebral Ultrasound

**DOI:** 10.1016/j.mcpdig.2024.09.003

**Published:** 2024-10-09

**Authors:** Tahani M. Ahmad, Alessandro Guida, Sam Stewart, Noah Barrett, Michael J. Vincer, Jehier K. Afifi

**Affiliations:** a Department of Pediatric Radiology, IWK Health, Halifax, Nova Scotia, Canada; b Department of Diagnostic Radiology, Dalhousie University, IWK Health, Nova Scotia, Canada; c Department of Radiology, University of Jordan, Amman, Jordan; d Biomedical Translational Imaging Centre (BIOTIC), Department of Diagnostic Radiology, Dalhousie University, Halifax, Nova Scotia, Canada; e Department of Diagnostic Imaging, Nova Scotia Health, Nova Scotia, Canada; f Department of Community Health and Epidemiology, Dalhousie University, Halifax, Nova Scotia, Canada; g Faculty of Computer Science, Dalhousie University, Halifax, Nova Scotia, Canada; h Division of Neonatal-Perinatal Medicine, IWK Health, Halifax, Nova Scotia, Canada; i Department of Pediatrics, Dalhousie University, Halifax, Nova Scotia, Canada

## Abstract

**Objective:**

To develop deep learning (DL) models applied to neonatal cranial ultrasound (CUS) and clinical variables to predict neurodevelopmental impairment (NDI) in very preterm infants (VPIs) at 3 years of corrected age.

**Patients and Methods:**

This is a retrospective study of a cohort of VPI (22^0^-30^6^ weeks’ gestation) born between 2004 and 2016 in Nova Scotia, Canada. Clinical data at hospital discharge and CUS images at 3 time points were used to develop DL models using elastic net (EN) and convolutional neural network (CNN). The models’ performances were compared using precision recall area under the curve (PR-AUC) and area under the receiver operation characteristic curve (ROC-AUC) with their 95% ci.

**Results:**

Of 665 eligible VPIs, 619 (93%) infants with 4184 CUS images were included. The CNN model combining CUS and clinical variables reported better performance (PR-AUC, 0.75; 95% CI, 072-0.79; ROC-AUC, 0.71; 95% CI, 0.67-0.74) in the prediction of positive NDI outcome compared with the traditional models based solely on clinical predictors (PR-AUC, 0.60; 95% CI, 0.52-0.68; ROC-AUC, 0.72; 95% CI, 0.68-0.75). When analyzed by the CUS plane and acquisition time point, the model using the anterior coronal plane at 6 weeks of age provided the highest predictive accuracy (PR-AUC, 0.81; 95% CI, 0.77-0.91; ROC-AUC, 0.78; 95% CI, 0.66-0.87).

**Conclusion:**

We developed and internally validated a DL prognostic model using CUS and clinical predictors to predict NDI in VPIs at 3 years of age. Early and accurate identification of infants at risk for NDI enables referral to targeted interventions, which improves functional outcomes.

The advancement in neonatal care significantly improved survival of very preterm infants (VPIs <31 weeks’ gestation), but neurodevelopmental impairment (NDI) remains prevalent.^1^^,^^2^ This encompasses cerebral palsy (CP), cognitive delay, language delay, deafness, or blindness. Approximately 10%-15% of VPI develop CP, 40% display motor deficits and around 30%-50% have cognitive delay.^1^ Cranial ultrasound (CUS) is the primary screening tool for brain injury related to prematurity.^3^

Traditionally, statistical methods have been used for outcome prediction in neonatal literature, often relying on CUS findings and clinical assessments during toddlers and preschool age. Early identification of at-risk infants allows for targeted interventions and rehabilitation programs with the potential to improve quality of life and optimize health care resource allocation.^4^ With the rise of precision medicine and artificial intelligence (AI), a key question is how AI can improve the accuracy and timeliness of neonatal outcome predictions. Unfortunately, the use of machine learning (ML) in this context is limited,^4^ and the application of deep learning (DL) and convolutional Neuronal Networks (CNN) to CUS images routinely performed during the neonatal period to predict NDI in VPIs remains unexplored.

The objective of this study was to use AI to identify VPIs at risk of NDI before hospital discharge for prognostication and to facilitate early referral to rehabilitation services. In this study, we trained DL models capable of analyzing CUS images and predicting NDI. We compared the after 3 prediction models of NDI:

• the first model applied CNN to CUS images alone;
• the second CNN model combined CUS images and clinical variables;
• the third model applied elastic net (EN) regression to clinical variables only.

## Patients and Methods

### Study Design and Population

The IWK Health Research Ethics Board approval was obtained (Project #:1025476) for this study. This is a retrospective single-center study of a cohort of VPI (22^0^-30^6^ weeks’ gestation) born in Nova Scotia between January 2004 and December 2016 and admitted to IWK Health. Only survivors who had a 36-month neurodevelopment assessment completed were eligible.

### Exclusion Criteria

The exclusion criteria were as follows:

• congenital anomalies or chromosomal aberrations;
• palliation at birth;
• missing serial CUS scans or outcome data.

### Source of Data and Data Collection

Clinical data were retrieved from the AC Allen Perinatal Follow-up Program (PFUP) Research Database, which prospectively collects information on maternal, perinatal, and neonatal status (candidate predictors) and neurodevelopment at 36 months’ corrected age (outcome), with consent from all parents or guardians. Infant sex was included as a clinical variable because male sex is independently associated with poor outcomes in preterm infants.^5^

**Figure 1:**
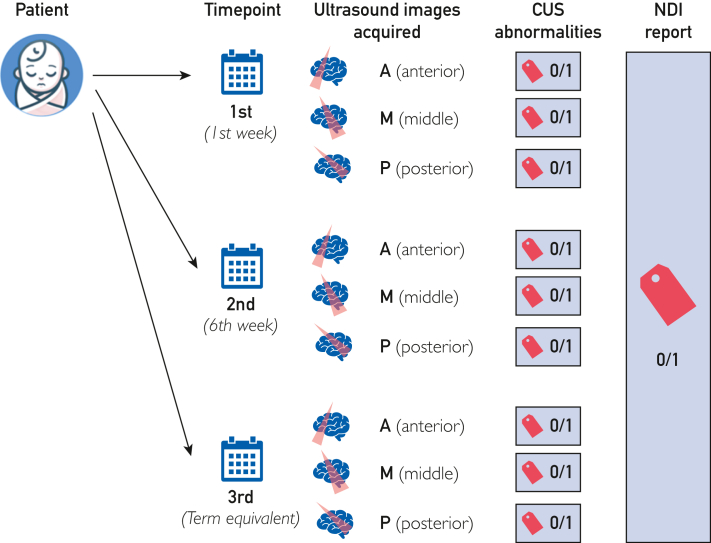
CUS data set schematic overview. For each infant, routine serial CUS screening was performed at 3 specific time points during the hospital stay. CUS images were retrieved from PACS, and 9 images per infant were collected to develop the CUS data set. Each image was manually curated by a pediatric radiologist who provided a binary classifier indicating the presence (1) or absence (0) of abnormalities on CUS. At 3 years corrected age, the neurodevelopmental outcome for each infant is classified as having NDI (1) or not having NDI (0). CUS, cranial ultrasound; NDI, neurodevelopmental impairment.

Ultrasound data were retrieved from the provincial Picture Archiving and Communication System (PACS). At IWK, CUS is routinely performed on all VPIs on at least 3 time points: at first and sixth weeks of life and at near-term age. Three coronal images were selected at each time point at preidentified anatomical landmarks including the following: A, anterior image at the level of the foramen of Monro; M, middle image at the level of the choroid plexuses and body of lateral ventricles; P, posterior image at the posterior periventricular white matter. Hence, the complete set yields 9 images per infant (Figure 1). The CUS images were deidentified and encoded. The clinical data and CUS images were linked using a unique identifier for each infant.

### Neurodevelopmental Assessment

All VPIs are regularly assessed by a multidisciplinary team at the PFUP. This includes the Bayley Scales of Infant and Toddler Development (Bayley third edition),^6^ ophthalmologic, auditory, and neuromotor examinations. When CP is diagnosed, grading is on the basis of the Gross Motor Functional Classification System.^7^ NDI is defined as any of CP, Bayley scores of <1 SD on any developmental domain, blindness, or deafness.

### Data Preparation and Processing

#### Clinical Variable Preprocessing

Clinical variables were grouped into 3 tiers*:* prenatal (maternal and pregnancy), perinatal (delivery and early postnatal), and neonatal variables (during the entire hospital stay) (Table 1). Missing values in the clinical data set were imputed with the variable median value; ordinal variables were converted to numerical variables maintaining their cardinality, and categorical variables were preprocessed using 1-hot encoding. Finally, numerical variables were normally scaled with z-score normalization.

**Table 1:** Population Characteristics

	Missing	Overall (n=619)	NDI (n=178)	No NDI (n=441)
Prenatal variables				
Maternal age (y), mean ± SD		29.7±5.6	28.6±5.8	30.1±5.5
Single family composition	26 (4)	76 (12.3)	30 (16.9)	46 (10.4)
Hollingshead socioeconomic status				
Class I	75 (12.1)	59 (9.5)	12 (6.7)	47 (10.7)
Class II		195 (31.5)	42 (23.6)	153 (34.7)
Class III		150 (24.2)	36 (20.2)	114 (25.9)
Class IV		96 (15.5)	38 (21.3)	58 (13.2)
Class V		44 (7.1)	24 (13.5)	20 (4.5)
Maternal diabetes		576 (93.1)	160 (89.9)	416 (94.3)
Maternal hypertension		117 (18.9)	29 (16.3)	88 (20.0)
Chorioamnionitis/funisitis		84 (13.6)	30 (16.9)	54 (12.2)
Perinatal variables				
Optimal antenatal steroids		208 (33.6)	44 (24.7)	164 (37.2)
Intrapartum magnesium sulfate		249 (40.2)	61 (34.3)	188 (42.6)
Delivery room resuscitation (compression/epinephrine)		46 (7.4)	30 (16.9)	16 (3.6)
5-min Apgar score, mean ± SD	7 (1.1)	7.1±1.8	6.7±2.1	7.3±1.6
Infant male sex		339 (54.8)	111 (62.4)	228 (51.7)
Gestational age (wk), mean ± SD		27.9±1.8	27.3±2.1	28.1±1.7
Birth weight (g), mean ± SD		1137.5±315.8	1055.7±337.9	1170.6±300.6
Outborn status		46 (7.4)	16 (9.0)	30 (6.8)
Cesarean delivery		368 (59.5)	108 (60.7)	260 (59.0)
Postnatal variables				
Major operation		56 (9.0)	31 (17.4)	25 (5.7)
Patent ductus arteriosus		60 (9.7)	34 (19.1)	26 (5.9)
Neonatal systemic infections		180 (29.1)	71 (39.9)	109 (24.7)
Severe (grade 3,4) IVH		46 (7.4)	34 (19.1)	12 (2.7)
WMD (porencephaly or PVL)		29 (4.7)	25 (14.0)	4 (0.9)
Bronchopulmonary dysplasia	41 (6.6)	157 (25.4)	68 (38.2)	89 (20.2)
Mechanical ventilation (h), mean ± SD		320.9±569.3	586.3±801.8	213.9±395.8
Length of hospital stay (d), mean ± SD	11 (1.7)	82.2±50.8	105.0±77.6	73.0±30.2
Systemic steroids (dexamethasone)		101 (16.3)	51 (28.7)	50 (11.3)

Data are presented as number (percentage), mean ± SD, or median (IQR). Necrotizing enterocolitis (≥Bell stage II) and retinopathy of prematurity were dropped because of <5 infants’ strata.

IVH, intraventricular hemorrhage; NDI, neurodevelopmental impairment; PVL, periventricular leukomalacia; WMD, white matter disease.

#### Data Set Labeling

Two sets of labels were acquired (Figure 1) as follows:

• CUS label: All CUS studies reported as abnormal findings (abnormal CUS indicates the presence of any intracranial hemorrhage, white matter changes, hydrocephalus, or cerebellar injury) were reviewed, and each of the 9 selected coronal images was manually labeled by a single pediatric neuroradiologist (T.A.; >10 years’ experience). To validate the process, the radiologist also examined 40 randomly selected CUS reported as with normal findings to confirm the accurate labeling. The CUS label was used in our previous work to train a diagnostic model to predict abnormal CUS (encoded as 1) or normal/unremarkable (encoded as 0).^8^ It is reported in this work as a reference for discussing modeling challenges (see Discussion and Supplemental Table 3, available online at https://www.mcpdigitalhealth.org/).
• Outcome (NDI label) reflects the neurodevelopmental outcome for each VPI at 3 years as extracted from the PFUP database with 1 indicating NDI and 0 denoting no NDI. This label is the main label used to train the prognostic model described in this article.

#### Image Data Preprocessing

Each CUS image underwent anonymization, coding, and linking with clinical data set, followed by conversion to RGB color space using the *pydicom* library and rescaling to 224 × 224 resolution.^9^ A region of interest mask was applied using Label Studio.^10^ Transfer learning with ImageNet,^11^ and data augmentation techniques were used to address the sample size and class imbalance. Various deep neural network architectures were tested through an iterative approach identifying EfficientNetB0^12^ as the most performing network for our specific task. It was implemented in Python and PyTorch (version 1.12) and trained using the Adam optimizer and a 0.001 learning rate. GRADCAM heatmaps^13^ were applied to abnormal findings on CUS images to highlight areas influencing model prediction, aiding health care providers in discerning the abnormal areas on the image (Supplemental Figure 1, available online at https://www.mcpdigitalhealth.org/). Further details of similar image processing and model development are also elaborated in our previous work.^8^

### Model Development and Evaluation

The data set was split 80:10:10 into training, developing, and testing sets, respectively, using stratified sampling, ensuring that CUS from the same infant were grouped, and the “outcome” label was evenly distributed across splits. To mitigate class imbalance, we used minority class oversampling. The test set was sampled to have the outcome class as close as possible to 50%. This process was repeated 10 times using random bootstrap sampling.

Multiple ML strategies were used to develop and test 3 models—model 1, a CNN model using only CUS images; model 2, a CNN model combining CUS with clinical predictors; and model 3, an EN model using only clinical predictors.

Precision recall area under the curve (PR-AUC) was chosen as the primary metric because of it being less affected by high true-negative ratios, common in imbalanced data sets. Area under the receiver operation characteristic curve (ROC-AUC) was also included for broader comparability with other studies because of it being widely used. Both metrics offer aggregated scores ideal for comparing model discrimination without requiring a predefined threshold for type I and type II errors.

#### Multi-input Network Strategy

We implemented a simple network that combines both clinical data and CUS images (model 2) for binary classification. Images are processed with the same pretrained CNN (from model 1, pretrained on ImageNet data^11^), whereas clinical data are processed through a multilayer perceptron. The outputs from both are combined and sent to a classifier layer. The design included Relu activation and dropout (Supplemental Table 1, available online at https://www.mcpdigitalhealth.org/).

#### Multiple Instance Learning Strategy

Multiple instance learning (MIL) strategy are applied to data sets characterized by 1 input mapping a group of instances (ie, serial CUS images per infant).^12^ This framework is often used for weakly supervised learning tasks, such as classifying histopathologic slices.^13^^,^^14^ The implementation used the MONAI framework,^15^ with EfficientNet-B0 as the backbone classification network and attention mechanism as the head.^16^

#### Specialized Model Strategy

As an alternative to model 1 (CNN trained with CUS only), we trained a single model on all time points and planes. Additionally, we trained 9 specialized models, each focused on a specific combination of plane and time point (Table 2).

**Table 2:** Prediction Models of NDI: Comparing Discrimination at Different Ultrasound Planes and Acquisition Time Points

NDI prediction metric	CUS plane	CUS acquisition time point		
		1	2	3
PR-AUC (95% CI)	Anterior	0.67 (0.50-0.86)	0.81 (0.77-0.91)	0.76 (0.66-0.87)
	Middle	0.68 (0.47-0.83)	0.76 (0.60-0.90)	0.77 (0.69-0.86)
	Posterior	0.67 (0.63-0.73)	0.63 (0.49-0.79)	0.66 (0.54-0.82)
ROC-AUC (95% CI)	Anterior	0.63 (0.52-0.81)	0.78 (0.66-0.87)	0.70 (0.64-0.83)
	Middle	0.66 (0.52-0.76)	0.74 (0.63-0.88)	0.70 (0.61-0.82)
	Posterior	0.62 (0.60-0.67)	0.61 (0.45-0.76)	0.58 (0.40-0.79)

Different from Table 2, this table summarizes the performance of 9 different specialized models (on the basis of model 1 from Table 2), trained on each combination of plane and time point. Each model is therefore trained only on a subset of the entire data set and can be used only on a specific plane and time point.

CUS, cranial ultrasound; NDI, neurodevelopmental impairment; PR-AUC, precision recall area under the curve; ROC-AUC, area under the receiver operating characteristic curve.

### Statistical Analyses

Only VPIs with clinical and CUS data were included in the analysis. The models’ predictive performance was compared using ROC-AUC and PR-AUC with their 95% CIs. Exploratory analysis evaluated NDI prediction models by CUS plane (anterior, middle, or posterior coronal planes) and acquisition time point (first week, sixth week, and term equivalent age).

For DL models, clinical variable importance was assessed using feature importance from random forest, ranking the most important features by Gini index and refining through recursive feature elimination. To compare the accuracy of DL models of CUS in predicting NDI, an EN model was trained using only clinical predictors (model 3). Hyperparameter optimization was conducted via random search 10-fold crossvalidation using Scikit-Learn’s EN module.^17^

## Results

**Figure 2:**
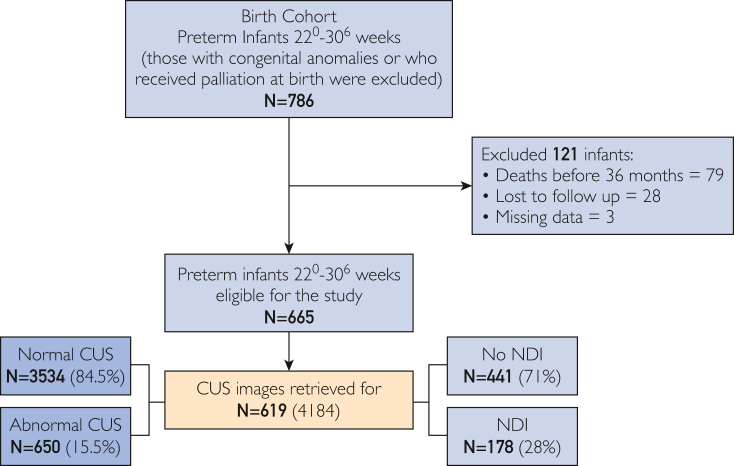
Population flowchart. CUS, cranial ultrasound; NDI, neurodevelopmental impairment.

Of 665 eligible VPIs, 619 (93%) were included in the analysis. The mean gestational age was 27.9±1.8 weeks, the mean birth weight was 1137±315.8) g, and 55% were male infants. In this cohort, 178 infants (29%) developed NDI, whereas 441 (71%) had no NDI (Figure 2). The population characteristics are summarized in Table 1. For the 619 VPI, 4184 images were retrieved; 84.5% of these CUS were classified as normal at all time points and 15.5% reported abnormal findings at any time point (Figure 2).

On internal validation, all 3 models provided good discrimination of VPI with and without NDI with comparable ROC-AUC and overlapped 95% CIs as shown in Table 3 and Supplemental Figure 2A (available online at https://www.mcpdigitalhealth.org/). In view of class imbalance in this cohort favoring the majority class (29% NDI and 71% no NDI), the PR-AUC (95% CI) focusing on positive prediction was compared between the 3 models (Supplemental Figure 2B, available online at https://www.mcpdigitalhealth.org/). Both CNN models of CUS, with and without incorporation of clinical variables, provided good predictive performance and were superior to EN trained solely on clinical variables (Supplemental Figure 2B, available online at https://www.mcpdigitalhealth.org/). Incorporating clinical predictors in the CNN model reported overall better performance, although not statistically significant, than the CNN model relying on CUS alone (PR-AUC, 0.75; 95% CI, 0.72-0.79; and PR-AUC, 0.73; 95% CI, 0.71-0.74, respectively). Although the EN model had a good overall ability to distinguish between positive and negative cases (Supplemental Figure 2A, available online at https://www.mcpdigitalhealth.org/), it reported a poor prediction of positive cases with NDI as shown by PR-AUC of 0.60 (95% CI, 0.52-0.68) (Supplemental Figure 2B, available online at https://www.mcpdigitalhealth.org/).

**Table 3:** Prediction Models of Neurodevelopmental Impairment in Very Preterm Infants: Comparing Predictive Performance

Model		Candidate predictors	Predictive performance	
Name	Strategy		PR-AUC (95% CI)	ROC-AUC (95% CI)
Model 1	CNN	CUS images only	0.73 (0.71-0.74)	0.68 (0.65-0.71)
Model 2	CNN	CUS + clinical variables	0.75 (0.72-0.79)	0.71 (0.67-0.74)
Model 3	Elastic Net	Clinical variables only	0.60 (0.52-0.68)	0.72 (0.68-0.75)

CNN, convolution neuronal network; CUS, cranial ultrasound; NDI, neurodevelopmental impairment; PR-AUC, precision recall area under the curve; ROC-AUC, area under the receiver operating characteristic curve.

In this cohort, on exploratory analysis focusing on specific CUS planes and time points, the best-performing prediction model of NDI was trained using CUS images only from the anterior plane at 6 weeks of chronological age with an ROC-AUC of 0.78 (95% CI, 0.66-0.87) and PR-AUC of 0.81 (95% CI, 0.77-0.91) (Table 2 and Supplemental Figure 3, available online at https://www.mcpdigitalhealth.org/).

## Discussion

This study explored different ML techniques to predict NDI in VPI using CUS images, clinical variables, or both. Our findings indicate that incorporating CUS images along with clinical predictors resulted in an effective model capable of predictive positive NDI outcomes, compared with models using only clinical variables. The EN model relying solely on clinical variables had a similar ROC-AUC to the combined CNN model but a significantly lower PR-AUC, highlighting its limitations in correctly identifying positive cases, despite reasonable overall classification performance. Notably, the CNN models reported the best performance, balancing positive and negative prediction of NDI, and provided the highest PR-AUC indicating superior performance in correctly classifying positive cases (infants with NDI) in the context of class imbalance.

The developed prediction models in this study provided good discrimination between VPIs with and those without NDI with an ROC-AUC and PR-AUC of >0.7. These findings align with previously published studies, although differences exist in predictors, outcomes, and the use of DL methods.^18^, ^19^, ^20^ Ambalavanan et al^18^ used multivariate logistic regression to predict death or NDI at 18-22 months in a cohort of extremely low birthweight infants using clinical predictors and ultrasound reports, reporting a c-statistic and misclassification rates of 0.68 and 0.32, respectively, for NDI among survivors.^18^ The study was limited by high loss to follow-up rates, use of an older cohort (1997-2005), inconsistent NDI ascertainment, and focus on early (7 days) CUS findings, mainly intraventricular hemorrhage, thus limiting its generalizability. Routier et al^19^ reported that a multimodal approach outperformed unimodal predictions of death or NDI at 24 months in extremely preterm infants (area under the curve, 0.91 vs 0.81, 0.77, and 0.79, respectively). This single-center study was limited by a small sample size (n=109 infants) and relied on early neonatal predictors during the first 2 postnatal weeks. Chung et al^20^ compared different ML methods to predict NDI at 24 months in a large cohort of VPI and achieved AUC values ranging from 0.73 to 0.83 but was limited by the use of late predictors (at 6 and 12 months of corrected age) including Bayley scores, relying on clinical variables and CUS reports.

Developmental outcomes at 24 months may not reliably predict long-term functional, educational, and social skills in preschoolers. Taylor et al^21^ found that many VPIs with moderate to severe NDI at 2 years reported mild or no impairment by age 10, highlighting the influence of environmental and rehabilitation factors on developmental trajectory.^21^ We predicted 3-year neurodevelopmental outcomes during the neonatal period, this is critical for wise allocation of health care resources, and early enrollment in rehabilitation programs during the window of neuroplasticity. Future longitudinal research of the preterm population should extend efforts to conduct developmental assessments at and beyond school age. Several challenges affecting the model’s performance were identified, related to intrinsic data set limitations and experimental design as described further.

### Intrinsic Data Set Factors

CUS images were acquired by various manufacturers and operators over several years, resulting in a heterogeneous data set susceptible to overfitting. Our previous work^8^ provides a detailed analysis of the impact of different vendors on the data. Simpler CNN models with fewer parameters performed best, effectively managing overfitting and generalizing better to new data.

An MIL strategy was initially adopted, but it performed poorly, with a PR-AUC (95% CI) of 0.57 (0.55-0.58) and ROC-AUC (95% CI) of 0.58 (0.54-0.60). To rule out methodologic issues, positive controls were included, confirming the model’s ability to learn at maximum performance a simple task in a few epochs. The poor performance of the MIL strategy might be attributed to the reduced training set size. Unlike the simple CNN strategy, the MIL strategy required feeding complete sets of 9 images per infant; unfortunately, only 2754 (65.8% of the image data set) of the images met this requirement. Because CUS images were aggregated by the infant, the final training sample size (306 infants, 49.4%) might not be sufficient to capture the data set heterogeneity

Using specialized models for each specific time point and CUS plane reported variable performance, with some instances outperforming others, particularly at the second time point and for the anterior and middle coronal planes (Table 2). These planes better displayed anatomical structures susceptible to preterm brain injury, such as intraventricular hemorrhage and periventricular leukomalacia. The second time point at 6 weeks of chronologic age might capture brain injuries better, as some pathologies, such as white matter changes and posthemorrhagic ventricular dilatation, may not appear during the first week. However, the 95% CIs were large and tended to overlap, showing no clear trends.

### Experimental Design Factors

The complexity of the objective task is highlighted by the Matthew correlation coefficient (−0.04) between CUS and NDI (Supplemental Table 3, available online at https://www.mcpdigitalhealth.org/), indicating clinical confounding variables: situations in which an infant having CUS abnormality but did not develop NDI or where no abnormality was identified on CUS at any time point, but the infant subsequently developed NDI. This aligns with previous studies showing poor ability of CUS to predict NDI in preterm infants compared with magnetic resonance imaging or general movement video analysis.^22^ Some factors might explain these findings: (1) discordant labels, where early CUS abnormalities resolved by later assessments, leading to favorable outcomes; (2) the heterogeneous nature of NDI, particularly some components such as language delay may not have CUS structural correlates.

Overall, simpler image-based models and strategies tended to better predict NDI in preterm infants, likely due to data set heterogeneity and strong class imbalance, with significantly fewer abnormal than normal CUS images and limited representation of a certain subset of pathologies. Increasing the sample size could help address these issues and improve model performance.

### Strengths and limitations

The strength of the study lies in its inaugural novice design comparing different ML strategies for outcome prediction, the relatively large sample size of VPI at risk of NDI, and the low loss to follow-up rate of this cohort. In addition, internal validity was ensured by the standardized routine CUS imaging protocols during the study period and the robust method for neurodevelopmental assessment on the basis of validated tests (Bayley III). This study also builds ML expertise at our institution and fosters collaboration among clinicians, researchers, and computer scientists guiding future DL research on CUS images beyond segmentation tasks.

However, this study has limitations, including its single-center design and lack of external validation, which affects generalizability. Retrospective data collection involved different ultrasound machine vendors and operators, causing variability, along with class imbalance and limited pathology subsets on CUS. Mitigations were implemented, but future work is needed for external validation and translating this work into clinical practice.

## Conclusion

We developed and internally validated DL models to analyze CUS images of VPI and predict their outcomes. In this cohort, DL models combining CUS with clinical variables outperform statistical models based solely on clinical predictors. Several factors were identified that affected the prediction model’s performance, which may aid future researchers conducting similar studies.

Achieving higher performance will require studies with larger cohorts and more data. Future research should integrate CUS or magnetic resonance imaging with clinical data to enhance learning capabilities. Although AI focuses on prediction over explanation, models may achieve reliable predictions without interpretability. Thus, further studies in the domain of model explainability will be required in future work.

## Potential Competing Interest

This work is solely submitted to Mayo Clinic Proceedings: Digital Health. None of the authors have any relevant conflict of interests to disclose.
