# Supplementary material for: Deep Learning Model for Predicting Neurodevelopmental Outcome in Very Preterm Infants Using Cerebral Ultrasound
Source: Mayo Clin Proc Digit Health. 2024 Oct 9;2(4):596–605. doi: 10.1016/j.mcpdig.2024.09.003 (PMC11975984; doi:10.1016/j.mcpdig.2024.09.003)
Supplement: Supplemental Tables and Figures [file mmc1.pdf]

Supplementary Appendix

Supplementary Figure 1: GRADCAM heatmap

A grayscale coronal ultrasound image demonstrates abnormal dilatation of the occipital horn of the left lateral ventricle with increased echogenicity of the adjacent white matter (circle) ***a*** *the GRADCAM heatmap shows the most intense red hues at the site of CUS abnormality (arrow)* ***b***

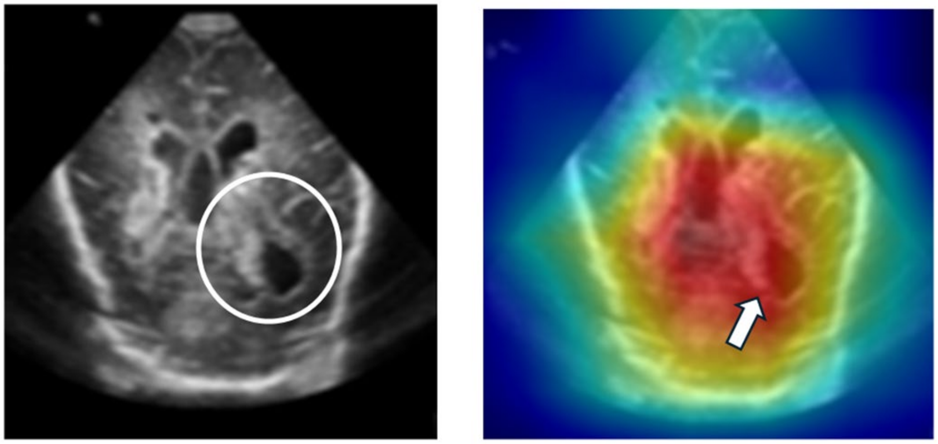

a b

Supplementary Figure 2 – Prediction Models of NDI: Comparison of Discriminative Performance

Model 1 (CNN trained on CUS images only); Model 2 (CNN trained on CUS images and clinical variables); Model 3 (Elastic Net trained on clinical variables only)

The plot on the left shows the ROC-AUC, while the plot on the right shows the PR-AUC.

Abbreviations: CNN = convolutional neuronal network, CUS = cranial ultrasound, NDI = neurodevelopmental impairment, PR-AUC = Precision-Recall Area Under the Curve, ROC-AUC = Area Under Receiver Operating Characteristics Curve.

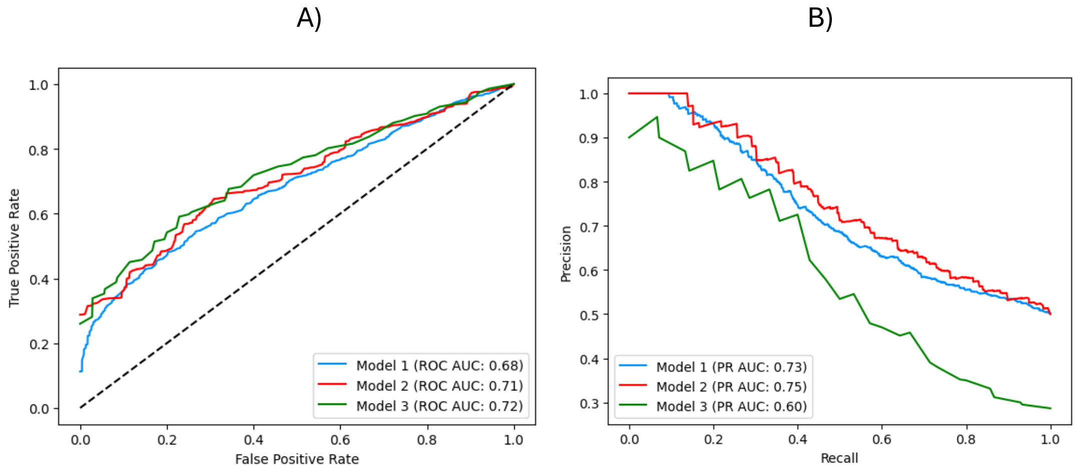

Supplementary Figure 3 – Prediction Models of NDI: Comparing Discrimination at Different CUS Planes and Acquisition Time points.

Abbreviations: PR-AUC = Precision Recall Area Under the Curve, ROC-AUC = Area Under Receiver Operating Characteristics Curve.

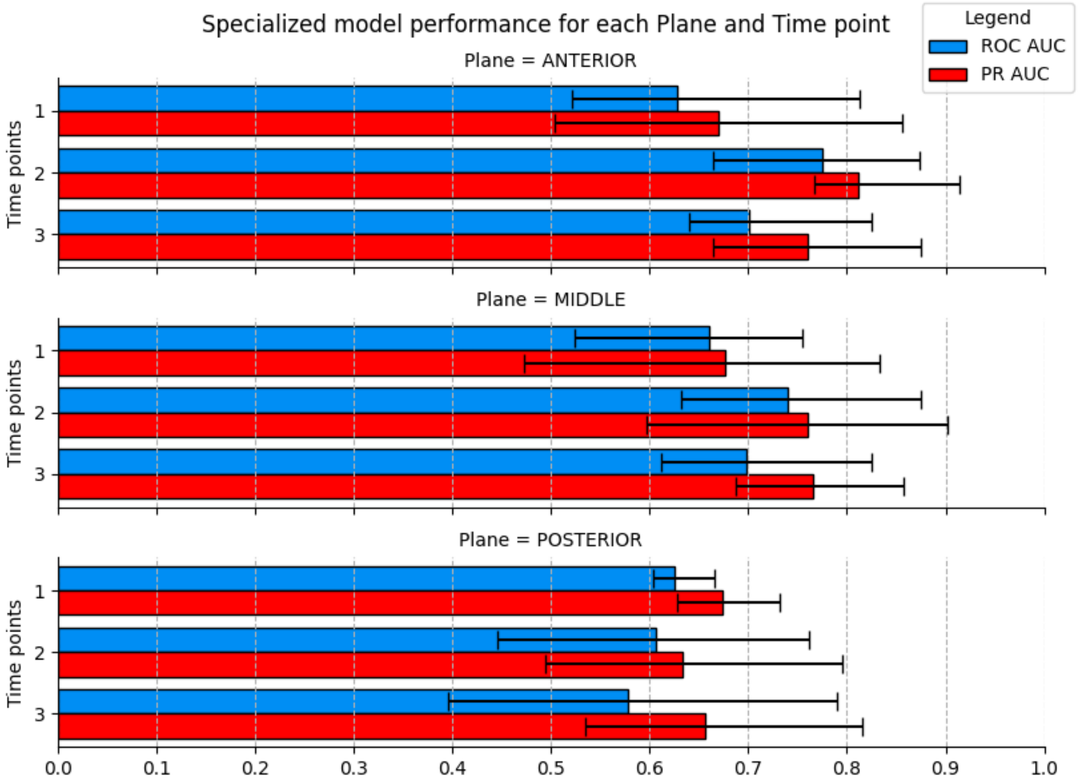

Supplementary Table 1 - Multi Input Network architecture.

Structure of the multi-input network with tensor output shapes used to train model 2 by combining CUS images and clinical variables.

<table>
<tr><td>Image input:
(batch, 3, 224,224)</td><td>Clinical variable input:
shape (batch, n variables)</td></tr>
<tr><td>pre-trained EfficientNet v2
(batch, 1280, 7, 7)</td><td rowspan="4">Linear layer
(batch, 64)</td></tr>
<tr><td>Global average pooling
(Batch, 1280, 1, 1)</td></tr>
<tr><td>Flatten layer
(batch,1280)</td></tr>
<tr><td>Relu activation
(Batch, 1280)</td></tr>
<tr><td>Dropout layer
(Batch, 1280)</td><td>Relu activation
(batch, 64)</td></tr>
<tr><td>Linear layer
(Batch, 64)</td><td>Linear layer
(Batch, 64)</td></tr>
<tr><td colspan="2">Concatenation layer
(Batch, 128)</td></tr>
<tr><td colspan="2">Relu activation
(Batch, 128)</td></tr>
<tr><td colspan="2">Classifier (Linear layer)
(Batch, 2)</td></tr>
</table>

Supplementary Table 2 – Descriptive Statistics of CUS Imaging Dataset

Our dataset is created by two sources: clinical and imaging datasets. The imaging dataset includes CUS acquired at different planes and time points and the clinical dataset classified outcome for each infant based on presence or absence of NDI. For each CUS image, a curation was performed identifying normal vs abnormal images. The following table presents a general overview of the number of CUS images assigned in each group (plane, and time point).

CUS Plane	Acquisition Time point	Neurodevelopmental Outcome		CUS Classification	
		**No NDI**	**NDI**	**Normal**	**Abnormal**
Anterior	1	330	127	385	72
	2	315	118	382	51
	3	277	115	342	50
Middle	1	411	168	456	123
	2	398	160	453	105
	3	361	156	463	54
Posterior	1	326	121	353	94
	2	310	111	358	63
	3	271	109	342	38
		(72%)	(28%)	(84%)	(16%)

Abbreviations: CUS = cranial ultrasound, NDI = neurodevelopmental impairment.

Supplementary Table 3 – Confusion Matrix of CUS and NDI

The confusion matrix of Ultrasound and NDI report labels from the ultrasound dataset shows Matthews Correlation Coefficient of -0.04. This suggests that the radiological interpretation of CUS alone is not a good predictor of NDI outcomes and highlights the complexity of the objective task in this project.

	**Neurodevelopmental Impairment**		
**Cranial ultrasound abnormality**		Negative	Positive
	Negative	2652	347
	Positive	882	303
